# General practitioners’ assessment and management of chronic kidney disease in older patients- a mixed methods study

**DOI:** 10.1186/s12875-024-02559-2

**Published:** 2024-08-20

**Authors:** Michelle Guppy, Esther Joy Bowles, Paul Glasziou, Jenny Doust

**Affiliations:** 1 Institute for Evidence-Based Healthcare, Faculty of Health Sciences & Medicine, Bond University, Gold Coast, Qld 4226 Australia; 2 School of Rural Medicine, University of New England, Armidale, NSW 2350 Australia; 3 Institute for Evidence-Based Healthcare, Faculty of Health Sciences & Medicine, Bond University, Gold Coast, Qld 4226 Australia; 4 Australian Women and Girls Health Research (AWaGHR) Centre, School of Public Health, Faculty of Medicine, The University of Queensland, Herston, Qld 4006 Australia

**Keywords:** Chronic kidney disease, Ageing, General practice, Continuity of care, Multimorbidity

## Abstract

**Background:**

Chronic kidney disease (CKD) is commonly managed in general practice, with established guidelines for diagnosis and management. CKD is more prevalent in the older population, and is associated with lifestyle diseases as well as social deprivation. Older patients also commonly experience multimorbidity. Current CKD guidelines do not take age into account, with the same diagnostic and management recommendations for patients regardless of their age. We sought to investigate general practitioners’ (GPs’) approach to older patients with CKD, and whether their assessment and management differed from guideline recommendations. We explored the reasons for variation from guideline recommendations.

**Methods:**

This was a mixed methods study of Australian GPs. An online anonymous survey about the use of CKD guidelines, and assessment and management of CKD was sent to 9500 GPs. Four hundred and sixty-nine (5%) of GPs responded, and the survey was completed by 399 GPs. Subsequently, 27 GPs were interviewed in detail about their diagnostic and management approach to older patients with declining kidney function.

**Results:**

In the survey, 48% of GPs who responded found the CKD guidelines useful for diagnosis and management. Four themes arose from our interviews: age-related decline in kidney function; whole person care; patient-centred care; and process of care that highlighted the importance of continuity of care. GPs recognised that older patients have an inherently high risk of lower kidney function. The GPs reported management of that higher risk focused on managing the whole person (not just a single disease focus) and being patient-centred. Patient-centred care expressed the importance of quality of life, shared decision making and being symptom focused. There was also a recognition that there is a difference between a sudden decline in kidney function and a stable but low kidney function and GPs would manage these situations differently.

**Conclusions:**

GPs apply guidelines in the management of CKD in older patients using a patient-centred and whole person approach to care. Older patients have a high prevalence of multimorbidity, which GPs carefully considered when applying existing CKD-specific guidelines. Future iterations of CKD Guidelines need to give due consideration to multimorbidity in older patients that can adversely impact on kidney function in addition to the expected age-related functional decline.

## Background

Chronic kidney disease (CKD) is an overarching diagnosis that defines disease as kidney function below an estimated glomerular filtration rate (eGFR) of 60 mL/min/1.73m^2^, or structural damage to the kidneys indicated by haematuria or albuminuria for more than 3 months [1]. Its reported prevalence is up to 10% of the population [2]. It has a strong association with older age, the prevalence of CKD over the age of 75 being 50% [3]. CKD is also associated with social deprivation and rurality [4]. Of additional concern, CKD is an independent risk factor for cardiovascular disease [5]. Cardiovascular disease risk increases with decreasing kidney function, and this is a continuum where there is no single cut-off value above which risk can be said to be higher [5].

Care in general practice is important for the identification and management of CKD [3]. In the United Kingdom (UK), to encourage the comprehensive care required to identify CKD, there is a CKD register and quality improvement funding to incentivise general practitioners (GPs) to aim for CKD management targets [3]. In Australia there is a CKD management handbook [6, 7] that is based off international guidelines [8], but no national register for CKD and no specific CKD financial incentives. Some beneficial evidence-based processes of care for CKD include: patient education to equip them to make better informed choices; GPs’ proactivity in addressing cardiovascular risk; and GPs supporting lifestyle change [3]. Additionally, improved quality of life and better blood pressure control can be achieved with tailored education provided to patients with CKD, and telephone guided access to community support [9]. Since cardiovascular disease management is an important component of care in CKD, GPs should be encouraged to use an absolute cardiovascular risk approach [10]. However, there is evidence that GPs are deviating from CKD clinical guidelines in Australia, the UK, Europe and elsewhere [11]. GPs are inconsistently using CKD registers, and providing what is considered to be suboptimal management [9]. They are also inconsistently making the diagnosis of CKD with their patients, citing concerns around diagnostic criteria and unnecessary labelling of patients with a disease for which progression to end-stage kidney disease is rare [11].

To address these inconsistencies, it is important to investigate how GPs are identifying, diagnosing and managing older patients with CKD in general practice, and to understand their clinical reasoning process and rationale for decision making [12].

## Methods

### Aim

Our study aimed to explore how GPs approach older patients with declining kidney function, the structure of these consultations, and their attitudes towards negotiating joint goals for care with patients. Our primary research questions were: How do GPs manage older patients with declining kidney function? How do GPs use CKD guidelines in their older patients with declining kidney function? Do GPs consider older patients differently to younger patients in the assessment and management of CKD? In our survey research we specifically referred to the Australian CKD management handbook (2015) [7]. In our interviews with GPs we used the term ‘guideline’ when discussing the evidence-based resources that GPs use to assist them in clinical decision making, and we gathered data on what resources they were using.

### Design and setting

An electronic questionnaire using the Qualtrics™ platform was sent via email to a random sample of Australian GPs through a third party database (Australasian Medical Publishing Company- AMPCo). The questionnaire asked GPs to self-rate their knowledge of CKD, their use of the CKD guidelines, and their experiences of managing patients with CKD in general practice. (See supplementary files).

At the conclusion of the questionnaire, GPs could indicate their willingness to be contacted to participate in an interview about how they manage CKD in general practice. Purposive sampling of GPs who responded was used to ensure a mixture of age, gender and geographic location of practice (urban, regional, rural, remote), spread throughout Australia. Recruitment of GPs continued until saturation of themes was reached. This study was approved by the Bond University Human Research Ethics Committee (approval number MG02860). Written informed consent was obtained from the GPs before the commencement of the interviews. A gift voucher of AUD$120 was given to GPs as an honorarium for their participation.

### Data collection and analysis

We aimed to survey 381 GPs based on a confidence interval of 95%, a margin of error of 5%, a population size of 30,000 and a conservative population proportion estimate of 50%. Survey responses were collected by the Qualtrics™ platform in mid-2018, and analysed in Microsoft Excel and SPSS v29 using descriptive statistics and Chi^2^ tests. Free text responses were analysed for themes. Interview questions were developed after consideration of the survey responses. The interview questions were piloted on two GPs and questions refined prior to participant recruitment. The interview questions are available in the Supplemental material. Interviews took place between May and July 2019, with a single interviewer (MG). MG is a female GP with a specialty qualification in general practice. A description of the researcher and research project were provided in the participant information statement prior to agreeing to interview, and reiterated at the commencement of the interviews.

Interview duration was up to one hour, and interviews were done either face-to-face, or online using the Zoom videoconferencing platform. GPs were at a location of their choice, usually either their clinic or home. Interviews were audio-recorded, and transcribed verbatim. Transcriptions were emailed to participants for review and correction, and then anonymised. NVivo version 12.0 was used to assist with the thematic analysis. Field notes were made by the interviewer immediately after the interviews, and shared with a second researcher (EJB).

The methodological framework used was reflexive thematic analysis, performed according to the method described by Braun and Clarke (2006) [13]. Two researchers (MG, EJB) familiarised themselves with the data as the interviews were proceeding. These two researchers independently generated initial codes. After several interviews had been completed, the researchers met to review the codes and agree on nomenclature and definitions. The researchers then independently completed the coding, and collated responses into themes. Further themes were refined by discussion and consensus in an iterative fashion.

## Results

Surveys were sent to 9500 GP email addresses, with 469 responses (5%), of whom 399 GPs completed the survey. Eighty-three GPs indicated they would be willing to be contacted for an interview. Of these GPs, 27 were interviewed. There were no drop-outs of interviewees. Of the GPs who responded to the survey, half were male and half were female, with age ranging from mid-20s to more than 80 years old. 74% of respondents had a specialty qualification in general practice. Participant demographic details for the survey and interviews are presented in Table 1, with a comparison to the Australian population of GPs. GPs were recruited by AMPCo from a range of geographic settings and proportionally across all the Australian states and territories. GP survey respondents were broadly similar to the Australian GP population in terms of gender and age range (Table 1.) For the interviews, we oversampled GPs in rural and remote, and the smaller states to ensure representation of opinion.

**Table 1:** Comparison of GP survey respondents (*n* = 399), GP interviewees (*n* = 27) to the Australian GP population (*n* = 39,259) [14, 15]

	GP survey respondents	GP interviewees*	Australian GP population
Age	≤ 39, 27% 40–49, 26% 50–59, 24% 60–69, 17% ≥ 70, 6%	≤ 39, 22% 40–54, 48% 55–64, 19% 65+, 4%	≤ 39, 25% 40–54, 37% 55–64, 21% 65+, 17%
Gender	Male 49% Female 51%	Male 40% Female 60%	Male 51%, Female 49%
Practice Location	Not collected	Urban 44% Non-urban 56%	Urban 68% Non-urban 32%
Australian State**	Not collected	NSW 22% QLD 22% VIC 19% WA 7% SA 7% TAS 11% ACT 4% NT 4%	NSW 24% QLD 15.5% VIC 19.5% WA 7% SA 5% TAS 1.5% ACT 1% NT 0.5%

*2 GPs did not state their age, **New South Wales (NSW), Queensland (QLD), Victoria (VIC), Western Australia (WA), Tasmania (TAS), South Australia (SA), Australian Capital Territory (ACT), Northern Territory (NT). NSW and VIC are the most populous states, and TAS, ACT and NT have the smallest populations

## Use of Australian CKD management handbook [7]

### Survey

From the results of the survey (*n* = 399), 67% of GPs were aware of the Australian CKD management handbook and 33% said they were not aware. There was no significant difference in terms of age of GP and awareness of the handbook (*p* = 0.99). There was also no significant difference in terms of length of practice and awareness of the handbook (*p* = 0.99). Overall, 48% of GPs found the CKD handbook useful for diagnosis and management. GPs reported that the most useful part of the handbook was the section on clinical action plans, with 68% reporting that was useful. A similar proportion (62%) found the summary to be most useful. Only 12% of GPs found the electronic apps useful. The majority of GPs (54%) were using the CKD handbook less than monthly, with 10% using them weekly, and 23% using them monthly. A minority proportion (14%) never used the CKD handbook. Only 19% of GPs had attended recent education (in the previous 6–12 months) on CKD.

### Interviews

In the interviews, we did not specifically refer to the Australian CKD management handbook, but asked questions about which evidence-based resources GPs were using. These included CKD handbooks and guidelines, cardiovascular disease guidelines, diabetes guidelines and other resources [1, 7, 16, 17]. We use the term ‘guideline’ to denote these resources. When discussing guidelines with the interview respondents, GPs were generally accepting of the CKD guidelines for care. Interview respondents fell into three categories of usage of guidelines: frequent users, infrequent users or non-users. There were only three respondents who were “never users” of the guidelines and they tended to be non-users of any clinical guidelines. These non-users were of the opinion that guidelines were lengthy and hard to follow, there were too many guidelines on numerous conditions, and instead they preferred to rely on their own clinical judgement. On the other hand, frequent users of clinical guidelines described them as easy to follow, user friendly, and a useful teaching tool for GP registrars. Infrequent users of the guidelines described using them for very specific actions, for example the “traffic light” flow chart. Or they reported that in the past they used to frequently use the guidelines, so now they were comfortable with managing patients without having to reference the guidelines every time. Many GPs preferred hard copies of the guidelines to online format.

### CKD risk assessment, diagnosis, and management in older patients

Four themes arose from the interviews around these topics: (1) Age-related change in kidney function, (2) Whole person care (3) Patient-centred care, and (4) Process of care in general practice. (See Table 2 for quotes relating to each theme).

**Table 2:** GP participant quotes pertaining to four identified themes

Theme One Age-related change in kidney function	
Increased prevalence	“*I’m much more cautious in older people*,* because you would expect them to have kidney problems by the fact that they are getting older*.” [GP8]
	“*Usually for patients more than 70 years old*,* we pretty much see as renal impairment*,* no matter how good their weight*,* their blood pressure and everything else is. So I just say that this is normal.”* [GP61]
Physiological change	“*Well a lot of older patients do have a deterioration of renal function anyway that could be just due to age-related changes in the blood supply*.” [GP2]
Diagnosis	“*I’d say*,* … the medical term is CKD which means chronic kidney disease … it’s what it’s called but the way I like to think of it … is that your kidneys aren’t working as well.”* [GP36]
Guidelines non-specific	*“I’m sure many GPs are concerned that there are so many tests and so much management*,* at what point do you continue this with someone who’s quite elderly*,* are we over testing or are we under testing?” [GP1]*
Risk management	*“Well yes it does I guess*,* simply because everybody’s eGFR goes down with age*,* … but I think that we don’t underestimate the importance of still managing that risk as well as you possibly can.”* [GP50]
	“*I guess because they are older and because my thinking is that something else may intervene and then sometimes when we get too active in our interventions*,* we start throwing lots of tablets and medications and things at people and that impacts on them*,* makes them more unwell really or not feel any better. So I’m probably more looking at quality of life*,* rather than just longevity*,* if you like*.” [GP25]
	“*I guess I have a higher tolerance for a lower GFR in those groups*,* it’s almost expected. But we can’t be complacent about it either*,* I understand it; it can be a sign of propensity to further significant kidney disease.*” [GP27]
Theme Two Whole Person Care	
Whole person	“*It’s not that the body is apart*,* but its combined*.” [GP8]
	“*I don’t focus on the kidneys per se. I focus on the entire health*.” [GP51]
Overlapping guidelines	“*I probably don’t specifically think about kidney disease is what I’m testing*,* I’d probably do it more in terms of cardiovascular disease*.” [GP25]
	“*Happily a lot of the management you do for a number of chronic conditions coincides nicely with the same for CKD*.” [GP27]
	“*There are so many guidelines*.” [GP5]
Balancing risks and harms	“*In the elderly you’ve got to be a little bit careful with overtreating (blood pressure)*” [GP1]
	“*Accepting that you may need to be a little bit circumspect*,* depending on if there are other comorbidities or frailties with the patient*.” [GP2]
Medication management	“*If they don’t have a life limiting illness and you’re just managing a lot of things*,* mental health*,* pain*,* decreased renal function*,* obviously I want to look after their kidneys so it’s always a factor in what I’m prescribing*” [GP51]
	“*I have a few older patients who are probably a little annoyed at me because I take them off their anti-inflammatories because I think it’s more important*,* but then they complain that their osteoarthritis has flared back up again*.” [GP66]
	“*I generally don’t find it too much of an issue to be honest. Most of the time you can easily juggle around…. multiple medications. You can easily juggle around or find alternatives*.” [GP75]
	“*Most of the time is monitoring. At other times is trimming out medications or even increasing some medications*,* depending*.” [GP16]
Theme Three Patient-centred care	
Shared-decision making	“*The first thing is what is the patient’s wishes*,* is it quality of life or do they want to live for as long as possible?”* [GP51]
	“*There’s a point where*,* you know*,* I suppose the life expectancy and the risk of life*,* you’re going on a preventative aim with your treatment and there’s a point where then you’re looking at trying not to cause harm and then there’s a point where you’re probably just trying to provide palliative care and quality of life. I suppose that is different for everyone*,* I think and it’s somewhat a decision between patients and doctors*.” [GP57]
	*“Often very elderly people aren’t that interested in heroic interventions and I have a chat with them about quality versus quantity of life and try and work out what is important with them and then go ahead accordingly with interventions or monitoring… I’m guided by them very much*.” [GP27]
Lifestyle change	“*You can’t change the mind of anyone in that age. They’ve got the good habits or they don’t*,* you know*,* no one suddenly becomes a gym junkie in their 70s*.” [GP51]
	“*We have got remarkable older patients*,* its just a matter of getting a shared goal*,* and I’ll often do- we have got amazing patients*,* I must say*,* who are really quite inspirational*.” [GP50]
	“*There are a fair few patients that I can think of whose social and yes*,* mental health and emotional issues far outweigh any physical issues they have for them*.” [GP36]
Quality of life	“*It probably comes down to looking at their general quality of life… I think it’s just probably encouraging them to live as well as they can*.” [GP50]
	“*I do lean on patient quality of life.”* [GP69]
	“*You look at their symptoms rather than their disease stats.*” [GP2]
Theme 4 Process of care	
Opportunistic vs. routine	“*If they come in for something else I take the opportunity.*” [GP30]
	“*I think you’re probably more likely to already have an eGFR on record.”* [GP1]
	“*We have a pretty formalised process…. Everyone gets a free shirt when they have an annual health check*,* so people come in for their shirt*.” [GP10]
Burden of care	“*Normally when you are busy and firefighting you don’t think about renal function*” [GP8]
	“*I think*,* as part of a general health review that we might do a little bit more often in older people*,* because we see them more often. We have the over-75 health assessment*,* which is another moment of time with patients. So I think it’s almost a more systematic approach and we’ve just got a - so I just do it as part of a list of a whole lot of other things.*” [GP22]
Referrals	*“It would worry me that if we referred everybody off in the categories where you need to refer off in elderly patients*,* it would swamp clinics that perhaps then means people who really need to be seen quickly by a nephrologist.” [GP1]*

#### Theme 1. Age-related change in kidney function

The expectation that older people were more likely to have kidney problems was a common sentiment. This was described in terms of “*higher risk*”, “*deterioration in renal function*”, “*everybody’s eGFR goes down with age*”. Many GPs emphasised this in terms of needing to be more careful about management of medications in older people and recognising older patients’ inherent increased cardiovascular risk. Two GPs used the term “*normal*” to describe this age-related decline in function, but only one GP described it as likely to be physiological [GP2]. In terms of making a diagnosis of chronic kidney disease with older patients, most GPs used a mixture of nomenclature. Many GPs described CKD in terms of reduced kidney function, and some did not use the term “CKD” (*n* = 7) when discussing this with patients.

Many GPs responded that the guidelines were non-specific with respect to older patients, and that this created uncertainty around outcomes. One GP felt that the criteria for chronic kidney disease “*didn’t strictly apply to older people*” [GP30]. This contrasted with many responses that kidney function in older patients should be considered the same as in younger patients. Many GPs expressed that risk management should still be considered important in older patients with respect to their morbidity and mortality. Some GPs had never thought of the guidelines applying to older patients differently. When it came to the discussion of management, several GPs felt there was less need to aggressively manage their kidney function. The sub-theme of balancing risk and harms was one that arose with considering how to manage older patients. This was predominantly discussed in terms of balancing medications that might improve or deteriorate kidney function. Ongoing monitoring of patients’ kidney function to ensure stability, or detect a problematic decline, was a common management approach.

#### Theme 2. Whole person care

A strong theme emerged that GPs considered the kidneys as part of the whole person, not just as a separate entity that could become diseased. GPs described considering the whole patient, their multiple medical problems, their functional status, and whether they had dementia or other significant health concerns as to how closely they would follow the CKD guidelines. This was a different but related concept to the third theme of patient-centred care, and so is described separately.

The theme of whole patient care, and not considering kidneys as a separate entity also emerged from use of guidelines. Many GPs did not consider kidney screening as a separate process, but as part of an overall cardiovascular risk assessment, or as part of the routine management of diabetes. Many GPs responded that they used alternate guidelines such as cardiovascular disease and diabetes management guidelines in preference to the kidney specific guidelines. These multiple overlapping guidelines covered similar clinical material. Examples included cardiovascular disease risk calculators online or within clinical software, locally developed online guidelines and other online resources.

The sub-theme of “balancing risks and harms” also came up many times with regard to whole person care, for example balancing the risk of a medication increasing falls while protecting the kidneys. GPs also described the balance between ensuring a patient understood the potential risks of chronic kidney disease versus not worrying them unnecessarily. A couple of GPs described that understanding the risks might be a motivating factor for patients to make lifestyle changes, but on the flipside did not want to create anxiety.

Medication management was an important sub-theme expressed by most GPs under the theme of “whole-person care”. This was discussed in terms of the synergistic use of medications to address multiple risks concurrently, and also the importance of considering nephrotoxic medications and deprescribing when there were contraindications, or medications were no longer necessary. Many GPs considered achieving the balance was often difficult due to an older patient’s needs for competing treatments. An example was the use of diuretics in heart failure, which might also overburden the kidneys. It was noted that if a person was under the care of several specialists, there could be competing priorities that were difficult to reconcile. This highlighted the burden of care on general practitioners to balance and adjudicate these priorities so as to provide the best quality of life for the whole person. Only one GP felt that this balance was not difficult to achieve. [GP75]

#### Theme 3. Patient-centred care

With respect to how the guidelines related to older patients a strong theme that arose was the concept of “patient-centred care”. GPs described a shared decision making approach and focusing on older patients’ concerns and expectations. This patient-centred approach to management was reflected in GPs’ responses to the well and healthy patient in addition to the patient with significant comorbidities. In healthy patients who had an unexpected or rapid drop in eGFR, GPs described investigating the patient to find out the cause, and manage accordingly. This was in contrast to patients in whom the decline in eGFR was either slow or stable, and patients who were already being monitored as part of whole person care of other comorbidities such as diabetes and cardiovascular disease. GPs described watchful waiting as a common management approach.

Most GPs felt there was benefit to lifestyle modification in patients at risk of CKD, but several expressed that lifestyle modification was difficult at any age. Most GPs would still counsel their older patients on smoking cessation, with one exception who felt they couldn’t change an older person’s mind. Some GPs were very optimistic about older patients’ capacity to change their lifestyle, with one GP describing the process as coming up with shared goals. Other GPs were realistic about patients’ situations, for example where mobility issues limit the ability to exercise effectively. Several GPs described the often complex social circumstances that patients were in (homelessness, relationship issues, food insecurity) and how this often took precedent over management of physical diseases. Another sub-theme that arose was “quality of life”, which was an important part of the shared decision making approach. GPs described quality of life in terms of encouraging patients to live as well as they can, focussing on managing their symptoms, and looking at what is the “*big picture*” for patients.

#### Theme 4. Process of care in general practice

The theme of “process of care” arose out of discussing risk assessment, and which we defined as the interventions and delivery processes occurring in the general practice setting. This included subthemes of organisational structures for risk assessment, use of medical software, burden of care, and relationships with secondary care. In discussion of risk assessment, the general approach could be described as either opportunistic or formalised/ routine. GPs who described an opportunistic approach explained that older patients saw the GP more frequently and so there was more of an opportunity to perform regular kidney function blood tests. Also, older patients were more likely to have had a blood test done for another reason. Other GPs described that when they were prescribing medications, either their medical software triggered a warning or they recognised themselves the need to consider the patient’s kidney status. Many GPs reported an approach that was individualised for each patient based on their family or personal history. GPs who used a more formalised or routine approach described supports for this approach. They included: government incentives for GPs to conduct health checks at specific ages; medical software reminders; and specific clinics that may have been led by nurses or GP registrars. Many GPs reported that they did formal screening for patients regarding diabetes or cardiovascular risk, and that kidney risk was considered part of this process. Others described specific screening groups and programs, particularly designed for Aboriginal and Torres Strait Islander patients.

We asked GPs what would be the trigger for them to input a diagnosis of chronic kidney disease into a patient’s record in their medical software. Most GPs said that they would put a diagnosis of CKD into their medical software for a “*low GFR*”, which in Australia is reported by laboratories as being < 60 mL/min/1.73m^2^. One of these GPs said they “*were on a crusade to clean up medical records*” [GP57]. Three GPs would not input the diagnosis until a lower GFR was determined (30–40 mL/min/1.73m^2^). Two said they “*didn’t worry about 60*” [GP 32, 62]. Four of the GPs were vague about whether they would put this diagnosis in their software, and three said they never did.

Another sub-theme expressed by a few respondents was the burden of care that general practitioners have to shoulder. They recognised that screening and management of risk was an ongoing and important part of general practice, but that it was often trumped by acute problems. Many GPs reported that the risk assessment performed in older patients used the same parameters as for younger patients. They described an increased frequency of screening in older patients, either because of routine screening, or opportunistic screening because older patients see the GP more often. GPs described that they considered their older patients to already be at higher risk of declining kidney function, and therefore potential kidney problems were on their radar.

With respect to management of older patients, several GPs described a change in how they referred older patients to specialist nephrologists compared to younger patients. They reported that they would continue to monitor a patient in general practice rather than referring them to a nephrologist. This prevented overwhelming elderly patients with specialty care and often unnecessary referrals, but also retained the burden of care for kidney management in general practice. GPs in rural and remote areas described the difficulty in accessing nephrologists, with the consequence being that they had little choice but to continue managing patients in general practice. In addition, they described the frustration rural patients felt at travelling long distances to see a nephrologist, often to have management continue unchanged by the nephrologist. This was in contrast to one urban-based GP who had a hospital and nephrology clinic directly across the road from their clinic, and described good access and communication with nephrologists.

## Discussion

Our study identified four major themes: (1) Age-related change in kidney function, (2) Whole person care, (3) Patient-centred care, and (4) Process of care in general practice. GPs recognised that older patients have an inherently higher risk of lower kidney function, and they managed that higher risk by managing the whole person and being patient-centred. In our study we found GPs were accepting of the term “CKD” but did not always use it when explaining the diagnosis to a patient. They tended to use descriptions around “reduced kidney function”. GPs varied on when they entered a diagnosis of CKD into the patient’s record in their medical software.

Whole person care recognised that many older patients have multimorbidity, and that dealing with the kidneys alone was “*a nonsense*”, particularly with respect to diabetes and hypertension, for which there are different and separate guidelines. Another aspect of whole person care was that recommendations from other specialists sometimes competed with CKD management. Patient-centred care expressed the importance of quality of life, shared decision making and being symptom focused. This took into account that some older patients with reduced kidney function are well and healthy, and that should be considered too. These patients could be more aggressively managed because they have a longer life expectancy. GPs also recognised that there is a difference between a sudden decline in kidney function and a stable but low kidney function, and reported that they would manage these situations very differently. Continuity of care tied together the theme of process of care in general practice. Patients were assessed for CKD either routinely or opportunistically, with a strong understanding that older patients were regular visitors to their GP.

The design of this study ensured that we sampled from a wide range of GPs around Australia. We had a variety of age, gender, and geographic spread of practice to ensure a representative sample. We had a low response rate to the survey, but the response rate achieved adequate power. Our quantitative data gives an understanding of the frequency of use of CKD specific clinical guidelines. With our interviews, we continued until saturation of data occurred. However, given the qualitative nature of the study we can’t draw conclusions about frequencies of perspective. We may have sampled GPs with a greater interest in CKD than the average GP population, given that our selection process required the completion of a survey and then further indication of an interest in participating in an interview. GPs with less knowledge of the CKD guidelines might have declined to be interviewed to avoid displaying any perceived deficits in knowledge.

### Prevalence of CKD in older patients

Kidney function declines with age, with the prevalence of chronic kidney disease as high as 50% of women over the age of 75 and 50% of men over 85 years under the current definition [3]. However, there is debate about the cause of this decline and whether it is associated with vascular comorbidity, or whether it primarily represents physiological ageing [3, 18]. Current Australian and international guidelines do not consider age, and have the same recommendations for younger and older patients [6, 8]. There is debate amongst nephrologists about whether older patients should be considered to have CKD under the current definition, or whether this age-related change in kidney function should result in an altered definition for older people [18–20]. GPs have also questioned the relevance of age, physiology and the interpretation of kidney function, and whether it is appropriate to label older patients as having CKD [21, 22]. GPs have expressed scepticism about the health benefit of this label for older patients [22]. Monitoring the trajectory of kidney function, and using age-related percentiles of kidney function to determine treatment decisions has been proposed [21, 23–25].

The GPs we interviewed recognised this increased prevalence of reduced kidney function in their older patients. A minority of GPs in our study described this as a normal physiological change. Moreover, it was reflected in the actions of a number of GPs who didn’t use the term CKD with their older patients, or a minority of GPs who didn’t put the term CKD into their medical software until the eGFR reached a level of 30–40 mL/min/1.73m^2^. The majority of GPs in our study accepted the guideline definitions of CKD in theory, and half said that they would treat older people the same as younger patients. However, in practice many took a patient-centred approach to care in applying the guidelines. GPs used monitoring as a common management tool, and described monitoring the trajectory of kidney function as a factor in decisions around patient treatment.

## Whole person care

Guideline deviation across multiple conditions is common in general practice [26]. Several studies describe a gap in GP adherence to CKD guidelines, poor recognition of CKD, and management that is suboptimal with low adherence to guidelines [3, 22, 27, 28]. GPs are reported as trivialising the diagnosis of CKD, and having insufficient knowledge of the clinical consequences of not treating CKD [22, 29].

Guidelines are seen as valuable by GPs to assist in reducing knowledge gaps and improving quality of care [22]. However, a critique of guidelines is that the context of the patient needs to be taken into account, and this is true in the context of the CKD guidelines. GPs described that single-disease guidelines cannot be applied simply to their patients with multimorbidity [28]. Multimorbidity is defined as the co-occurrence of two or more long term health conditions, and is very common with ageing [30, 31]. One in two people aged 65 years or over have multimorbidity [30]. Common co-morbidities include cardiovascular disease, diabetes, hypertension and atrial fibrillation, as well as mental health conditions [32]. Multimorbidity also includes frailty, sensory impairment (sight or hearing) and alcohol and substance abuse [33]. Both CKD and multimorbidity are also more common in areas of socio-economic disadvantage [30, 32]. Managing older patients involves significant complexity, taking into account social, cultural and economic factors as well as frailty, memory loss, cognitive impairment, patient passivity and lack of motivation [31]. Management of real patients is more complex than the information available in guidelines, and guidelines are not flexible enough to apply to individual patient circumstances [34]. For example, guidelines do not necessarily take into account patient frailty, physical limitations, care burden, and patient preferences [26].

GPs in our study did not explicitly use the term “multimorbidity”, but they did report on the multiple competing medical problems with their older patients. These GPs also discussed complex social and economic circumstances, frailty, physical limitations and competing priorities of care. GPs in our study described this complexity as a balancing act, particularly around medication management. This balancing act was a rationale for not always strictly following the single-disease focused CKD guidelines. Guidelines in older people should take multimorbidity into account. There is a UK National Institute of Clinical Excellence (NICE) guideline on multimorbidity that appropriately describes the management of older patients [33]. However, multimorbidity guidelines are not incorporated into CKD guidelines. GPs in our study articulated very clearly the differences between their healthy older patients and their frail older patients, and the different approach that they would take for both. It would be helpful if the guidelines provided more scope for this patient-centred approach to care.

### Patient-centred care

There is a tension between evidence-based outcome measures and patient-centred care [35]. GPs in the UK prioritise patient-centred care over prescribing guidelines when these two aims are considered not compatible [35]. One reason that GPs don’t follow guidelines is that guidelines don’t take into account individual patient needs [34]. Competing priorities in multimorbidity means CKD may be less of a focus. Addressing patients’ social and economic concerns often take priority. Issues to consider in a patient-centred approach to care include polypharmacy causing harm, and a high treatment burden affecting patient adherence [31]. Treatment burden includes time spent, cost, number of appointments and medications [36]. GPs and patients perceive that making lifestyle change requires considerable effort from the patient, and is not always achievable despite effort from the GP in attempting to motivate patients [37]. GPs propose the solution of relying on “*common sense*” to account for patient context and balance patient needs with evidence based recommendations [12, 38]. GPs’ clinical reasoning in complex situations and multimorbidity incorporates patient goals of care, preserves quality of life, as well as maintaining a balance between evidence-based care options [12, 39]. This is an ongoing and adaptive process requiring continuous review and prioritisation of the patient’s wishes [12, 39].

GPs in our study were mixed in their assessment of older patients’ capacity to change, citing some very motivated older patients, but also patients for whom lifestyle change was very difficult to achieve due to the patients’ circumstances. Many GPs in our study quoted quality of life as the main goal of treatment for older patients. Patient-centred decision making was a very common theme expressed by GPs in our study when considering management options. Their clinical reasoning approach included the balance between guideline-based recommendations, and the goals of quality of life and patient preferences.

### Process of care

Most CKD is managed in primary care [40]. Organisation of care and processes of care are an important factor in how CKD is managed in general practice. In the UK and Europe, governments have implemented ‘pay for performance’ targets where GPs are required to achieve evidence-based management targets for their patients [3, 28]. Australia does not have these programs and general practice in Australia is still fee-for-service, although it is moving to a patient enrolment model [41]. Structured approaches to care and quality improvement activities have been shown to improve health outcomes in the management of CKD [3, 40]. CKD requires complex time management which may not always be achievable in general practice [29, 31, 42].

However, the capacity for continuity of care in general practice has been a major factor in showing quality improvement of care in CKD [4]. Patients with a long-term relationship with their GP had greater success attaining blood pressure targets, and 87.3% of CKD patients in an Australian study had an annual eGFR performed despite formal registries not being in place [4]. Kidney function monitoring is problematic when patients do not have access to continuity of care [42]. Continuity of care has long been a key component of primary care and was originally defined as repeated contact between a patient and a doctor [43]. The definition has expanded recently to include the multidisciplinary team, with continuity of information provision and longitudinal understanding of patients using shared records [44]. Despite general practice becoming more complex and time-pressured, continuity is one of the most important aspects in managing multimorbidity and chronic disease [44]. Increased continuity of care is associated with increased patient satisfaction, increased medication compliance, reduced hospitalisation, and importantly, lower all-cause mortality rates [43, 45–48]. The longer a patient has the same GP, the greater the benefit of reduced mortality, and utilisation of acute services is also significantly reduced [48].

Another important aspect of continuity of care is to improve the primary-secondary care interface [45]. An enabler for quality care is clear and accessible referral pathways, and lack of access to specialty nephrology care is a barrier to management of CKD [29, 31, 42]. Communication between primary and secondary care is an important factor to improve patient care [31, 42]. This requires adequate information provision between specialists and generalists in both directions, and a shared understanding by providers of their roles in patient care, in order to coordinate and manage the burden of care for patients and clinicians [45].

GPs in our study had either opportunistic or formalised processes of care, the variation possibly reflecting the lack of government incentives for specific CKD care in Australia. They described a variety of experiences with referral to secondary care, generally reflecting issues around access to speciality care being reduced by geographic remoteness. Most reported that CKD management was very common in general practice, and that continuity of care (particularly with their older patients) allowed for regular review of patients even if a formal CKD management protocol was not in place. GPs acknowledged that there was a burden of care which was often trumped by the need to manage acute presentations. But nevertheless they recognised CKD management in older patients as an important component of general practice care.

The debate about age-related decline in kidney function is ongoing. GPs are caught in the middle of this debate, and the GPs in our study, whilst tacitly acknowledging the importance of guideline definitions, were in practice applying the guidelines to older patients in a patient-centred manner. Guidelines need to further consider management options around mild impairment of kidney function in older patients with multimorbidity. This is important in order to address the issues that GPs face of balancing risk with a patient-centred approach to care.

GPs should be seen as expert generalists, and this expertise not underestimated [29, 31, 38]. They have extensive experience and well formulated views on the application of single-disease guidelines to patients with multimorbidity [38]. GPs are deviating from guidelines because of their expertise around balancing treatment options, and their expertise in whole person and patient-centred care. Their expertise should be sought in the development of international guidelines [8]. Guidelines should be more integrated or cross-referenced (particularly CKD, diabetes and hypertension guidelines) [22]. There should be explicit acknowledgement in guidelines about the high prevalence of multimorbidity in older patients. Guidelines should include evidence from research on patients with multimorbidity [38]. Greater emphasis should be put on shared decision making with patients to achieve greater quality of life.

## Conclusion

GPs apply guidelines in the management of CKD in older patients using a patient-centred and whole person approach to care. Older patients have a high prevalence of multimorbidity, which GPs take into account when applying CKD-specific guidelines. Guidelines need to give due consideration to multimorbidity in older patients.

## Data Availability

Data are deidentified interview transcripts and survey responses, and are stored in a data repository at Bond University, Australia. Data are available on reasonable request by emailing researchdata@bond.edu.au.

## Abbreviations

NSW: Australian states- New South Wales
VIC: Victoria
Qld: Queensland
WA: Western Australia
SA: South Australia
TAS: Tasmania
ACT: Australian Capital Territory
NT: Northern Territory
CKD: Chronic kidney disease
eGFR/ GFR: (Estimated) glomerular filtration rate
GP: General practitioner
NICE: National Institute of Clinical Excellence
UK: United Kingdom
